# Automated Quantification of Pneumonia Infected Volume in Lung CT Images: A Comparison with Subjective Assessment of Radiologists

**DOI:** 10.3390/bioengineering10030321

**Published:** 2023-03-02

**Authors:** Seyedehnafiseh Mirniaharikandehei, Alireza Abdihamzehkolaei, Angel Choquehuanca, Marco Aedo, Wilmer Pacheco, Laura Estacio, Victor Cahui, Luis Huallpa, Kevin Quiñonez, Valeria Calderón, Ana Maria Gutierrez, Ana Vargas, Dery Gamero, Eveling Castro-Gutierrez, Yuchen Qiu, Bin Zheng, Javier A. Jo

**Affiliations:** 1 School of Electrical and Computer Engineering, University of Oklahoma, Norman, OK 73019-1102, USA; 2 School of Systems Engineering and Informatics, Universidad Nacional de San Agustín de Arequipa, Arequipa 04000, Peru; 3 Medical School, Universidad Nacional de San Agustín de Arequipa, Arequipa 04002, Peru

**Keywords:** infected lung segmentation, quantification of lung disease severity, comparison between manual and automated image segmentation, deep neural network, COVID-19 detection, COVID-19 severity assessment

## Abstract

Objective: To help improve radiologists’ efficacy of disease diagnosis in reading computed tomography (CT) images, this study aims to investigate the feasibility of applying a modified deep learning (DL) method as a new strategy to automatically segment disease-infected regions and predict disease severity. Methods: We employed a public dataset acquired from 20 COVID-19 patients, which includes manually annotated lung and infections masks, to train a new ensembled DL model that combines five customized residual attention U-Net models to segment disease infected regions followed by a Feature Pyramid Network model to predict disease severity stage. To test the potential clinical utility of the new DL model, we conducted an observer comparison study. First, we collected another set of CT images acquired from 80 COVID-19 patients and process images using the new DL model. Second, we asked two chest radiologists to read images of each CT scan and report the estimated percentage of the disease-infected lung volume and disease severity level. Third, we also asked radiologists to rate acceptance of DL model-generated segmentation results using a 5-scale rating method. Results: Data analysis results show that agreement of disease severity classification between the DL model and radiologists is >90% in 45 testing cases. Furthermore, >73% of cases received a high rating score (≥4) from two radiologists. Conclusion: This study demonstrates the feasibility of developing a new DL model to automatically segment disease-infected regions and quantitatively predict disease severity, which may help avoid tedious effort and inter-reader variability in subjective assessment of disease severity in future clinical practice.

## 1. Introduction

Computed tomography (CT) is the most popular medical imaging modality used in clinical practice to detect lung diseases (i.e., lung cancer, chronic obstructive pulmonary disease, interstitial lung diseases, pneumonia, and others). To more accurately assess the severity of many lung diseases and predict patients’ prognosis, estimation of disease-infected volume and/or its percentage to the total lung volume plays an important role. However, subjective estimation of disease-infected regions or volume by radiologists is quite difficult, tedious, and inaccurate (due to the large intra- and inter-reader variability), which makes it often infeasible in busy clinical practice. Thus, to help solve this clinical challenge, developing computer-aided detection (CAD) schemes or methods has been attracting broad research interest. For example, the CAD-generated lung density mask has been well developed and tested to quantify percentages of emphysema-infected lung volume [1] or degree of lung inflammation [2]. However, quantifying other lung diseases, such as the pneumonia-infected lung volume, has not been well developed and evaluated. Thus, we propose to investigate the feasibility of developing new CAD schemes that can automatically segment pneumonia-infected regions depicted on CT image slices and quantify the percentage of the diseased lung volume, which has the potential to assist radiologists in more accurately and efficiently reading and interpreting chest CT images in diagnosis of pneumonia-infected disease diagnosis and assessment of its severity.

In the last 3 years, SARS-CoV-2 virus named COVID-19 has infected millions of people globally [3] and it produces pneumonia-type diseases. Chest X-ray radiography and CT are two imaging modalities to assist diagnosis of COVID-19 induced pneumonia and/or monitor its severity [4]. While chest X-ray images are easier and faster to take, with lower cost, the CT scan is highly preferred mainly due to its three-dimensional nature and additional information to improve diagnostic accuracy [5,6]. Due to the wide and rapid spread of the COVID-19 virus, a large volume of chest X-ray images including CT images have been acquired in clinical practice. Meanwhile, several research image datasets with manual annotation masks have also become publicly available for researchers to develop new CAD schemes aiming to assist radiologists in more accurately and efficiently reading chest CT images to detect and diagnose COVID-19 induced pneumonia.

Recently, in developing CAD schemes of medical images, deep learning (DL) models have been well recognized and widely used to perform the tasks of segmenting the disease-infected regions of interest (ROIs) [7,8] and detecting or classifying diseases using the automatically extracted image features [9,10]. In using COVID-19 image datasets to develop CAD schemes, most of the previous studies focused on developing DL models to detect COVID-19 cases or classify between the COVID-19 and normal or other types of pneumonia cases [11,12,13,14]. Although many previous studies reported the extremely high accuracy of using DL models to detect and/or classify the COVID-19 infected cases (i.e., ranging from 90–100% accuracy [15]), no previous DL model is robust and clinically acceptable due to training bias and a “black-box” type approach [16]. Thus, the motivation of this study is to overcome disadvantages of previous DL models and investigate how to optimally use DL models to assist radiologists through increasing their accuracy and efficiency of disease diagnosis in future clinical practice. For these purposes, we propose a hypothesis that, in the technology aspect, it is important to add an interactive graphic user interface (GUI) to the DL model as a visual aid tool to increase the transparency of the DL model and allow radiologists to visually inspect results of DL model-segmented infected lesions or regions. In this application aspect, it is important to perform more observer performance or preference studies using DL models, which can help researchers better understand how to optimally develop and apply DL models to the future clinical practice to assist radiologists.

The objective of this study is to test our hypothesis. The study includes three steps or procedures. First, we build a novel ensembled DL model implemented with an interactive GUI to segment pneumonia-infected disease regions. Second, we conduct an observer reading and preference study that asks radiologists to estimate percentages of disease-infected volumes, assess disease severity, and rate their acceptance level for DL-generated lesion segmentation results. Third, we perform data analysis to compare agreement between the DL model and radiologists in the disease-infected region segmentation and disease severity assessment. The details of our study methods and results followed by discussions and conclusions are reported in this article. Specifically, Section 2 describes study datasets and the details of study methods to build a new DL model with a GUI tool and conduct the proposed observer study and data comparison analysis. Section 3 reports and explains study results. Section 4 discusses the unique characteristics or novelties and new observations or contributions of this study, as well as the limitations. Second 5 concludes this study and provides the take-home messages to the readers of this article.

## 2. Materials and Methods

### 2.1. Datasets

In this study, three chest CT image datasets were used, which include two public datasets, namely, “COVID-19 CT scans” and “COVID-19 CT segmentation dataset “https://www.kaggle.com/andrewmvd/covid19-ct-scans (accessed on 17 May 2021)”. The first public dataset includes 20 CT scans of patients diagnosed with COVID-19 from two sources, Coronacases “https://coronacases.org/ (accessed on 17 May 2021)” and Radiopedia “https://radiopaedia.org/ (accessed on 17 May 2021)”. Although numerous COVID-19 image datasets are publicly available, one unique characteristic of the datasets selected in this study is that all CT images have been annotated by experts providing three separate masks for the left lung, right lung, and infection regions. The second public dataset contains 100 axial CT images acquired from more than 40 COVID-19 patients. A mask with three labels is provided by a radiologist for each CT image indicating ground-glass opacity (GGO), pleural effusion and consolidation regions. These two datasets were used to build and/or train the DL model of segmenting and qualifying the disease infected regions or volumes. Additionally, another independent testing dataset including 80 CT scans of COVID-19 patients acquired from “Hospital Regional III Hanorio Delgado” Arequipa, Peru, was also assembled. This dataset is used to test and evaluate the trained DL models and conduct the proposed observer reading and preference study.

### 2.2. Image Preprocessing

To achieve higher reliability or robustness of the DL model, several image preprocessing techniques were employed to initially remove clinically unrelated images and normalize the remaining images. First, the “COVID-19 CT scans” dataset includes whole CT images of COVID-19 patients. However, some slices of each CT scan (i.e., in the beginning, and near the end of scan) usually contain very little lung area, thus not providing helpful information. Including these CT slices in the training data leads to a more unbalanced dataset. Thus, we removed up to 10% of CT images at the beginning and near the end of each CT scan. Generally, all lung infection datasets are unbalanced since the number of infection mask pixels is significantly less than the pixels of the healthy lung and other normal tissues presented in the image. To create a more balanced training dataset, we removed all healthy CT slices with no infection mask.

Second, since image normalization or standardization has been considered as an important preprocessing step when training deep neural networks to achieve high robustness or scientific rigor [17], we normalized all CT images by clipping the intensities outside the range [–1024, 600] HU. Specifically, if x > max, x’ = max, if x < min, x’ = min, and the remaining values are scaled between zero and one using a linear mapping equation: x’ = (x-x_min_)/(x_max_-x_min_).

**Figure 1:**
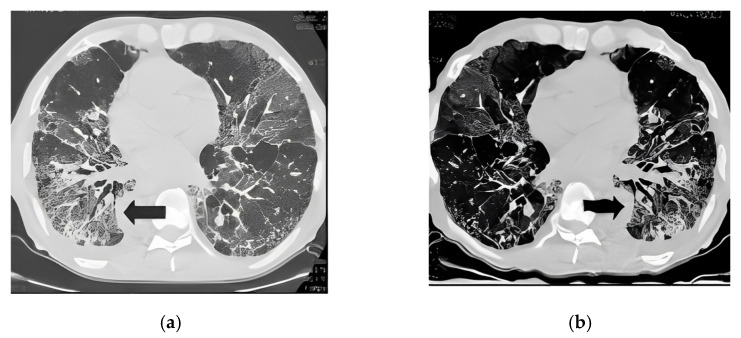
An example of applying an augmentation method. (**a**) The original image; (**b**) After applying an augmentation method.

Third, we applied the data augmentation technique to generalize and enlarge the dataset and mitigate overfitting. The main augmentation method adopted in this study is Elastic Transform [18] which is commonly applied in biomedical image analysis. The python library Albumentations [19] was used to perform the Elastic Transform and other affine transformations. Along with the elastic Transform, we also applied other common methods of horizontal and vertical flipping and random rotation to increase the size of training images. Figure 1 demonstrates the changes in a CT slice after applying an augmentation method in this study.

**Figure 2:**
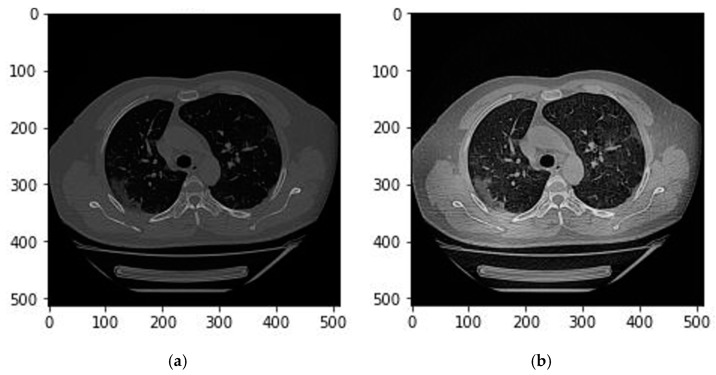
(**a**) Before applying a CLAHE filter; (**b**) After applying a CLAHE filter.

Last, we applied another image preprocessing technique using several filters to further enhance image features detected on the CT image. In this step, several filters have been tested with various channel arrangements to enhance different textures and structures and consequently achieve better discrimination between healthy and infected regions. For example, contrast Limited Adaptive Histogram Equalization (CLAHE) is one of the filters that has been applied as a channel to the CT images. CLAHE is a variant of adaptive histogram equalization that limits contrast amplification to reduce noise amplification. This filter performs histogram equalization in small patches with high accuracy and contrast limiting. Figure 2 illustrates the effect of applying a CLAHE filter on a CT image.

### 2.3. Image Segmentation Models and Output

Several common deep neural network models were selected and used in this study, including UNet [20], Feature Pyramid Network (FPN) [21], and Attention Residual UNet (AR-UNet) [22]. The Segmentation Models library [23] available on GitHub was also used to test various segmentation models with different backbones and parameters more conveniently. For each model, many parameters have been tested and modified, including loss functions, fixed and variable learning rates, encoders and decoders, and dropout rates.

#### 2.3.1. Lung Segmentation

**Figure 3:**
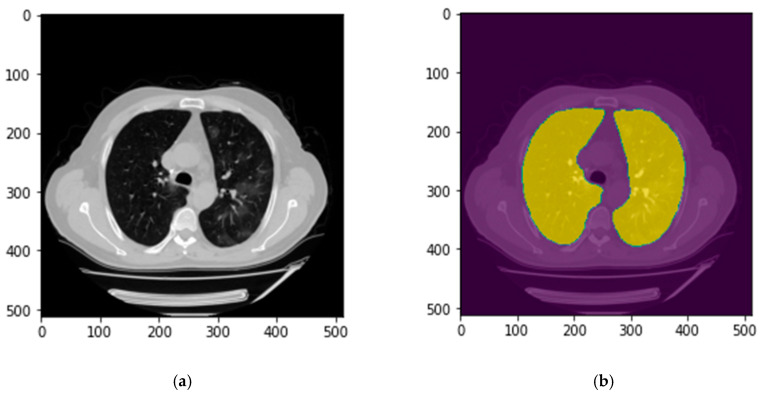
An example of the lung segmentation. (**a**) Raw CT image; (**b**) CT image and lung mask.

The first step is to segment the lung area depicted on each CT slide. For this purpose, a publicly available model for lung parenchyma segmentation was used to create lung masks and segment the lung area [24]. In brief, this model used the UNet, with the only adaption being batch normalization after each layer. Figure 3 demonstrates an example of the created lung mask and the lung segmentation result using this mask.

#### 2.3.2. Infection Area Segmentation

The next step is to segment the disease infected lung regions (from fuzzy ground glass to consolidation patterns). For this purpose, various object detection and segmentation models with different hyper-parameters have been tested and employed to achieve the highest accuracy. First, the AR-UNet is selected to build the ensembled model in this step. AR-UNet model is an end-to-end infection segmentation network, which embeds an attention mechanism and residual block simultaneously into the UNet architecture. Hence, this model efficiently balances the limited training data. In this model, the attention path employs the attention mechanism to capture spatial feature details. The residual block involves the semantic information flow through a 1 × 1 convolution [25].

Based on the literature search and our experiments, we recognize that among many tested loss functions, the Binary cross-entropy loss and the Tversky loss [24] led to the best predictions. Binary cross-entropy is calculated as the following Formula (1) [26].

(1) $$L_{BCE}=-\overset{2}{\underset{i=1}{\sum }}t_{i}\log\left(p_{i}\right)$$

where *t_i_* is the truth value (either 0 or 1), and *p_i_* is the SoftMax probability for the *i*th class.

To compute the Tversky loss function, a SoftMax along each voxel is applied [24]. Let P and t be the predicted and truth binary labels, respectively. The Dice similarity coefficient (D) between two binary volumes is identified and computed using Formula (2):

D (P, t) = 2|Pt|/(|P| + |t|)(2)

Since, in most cases, non-lesion voxels outnumber the lesion voxels, one of the main challenges in medical imaging is imbalanced data, especially in lesion segmentation. Therefore, using the unbalanced data in training lead to predictions that are severely biased towards low sensitivity (recall) and high precision, which is not desired, particularly in medical applications where false-positive (FP) detections are much more tolerable than false negatives (FNs). To achieve an optimum balance between sensitivity and precision (FPs vs. FNs), we used a loss layer based on the Tversky index. This index allows us to put emphasis on FNs and leads to high sensitivity. Using the formula (2) in a training loss layer, it equally weighs recall and precision, FN and FP, respectively [24]. To weigh FNs more than FPs in the training of a network with highly imbalanced data where small lesions’ detection is essential, a loss layer based on the Tversky index is efficient. The Tversky index is computed as the Formula (3) [24]:

Ti(P,t,α,β) = |Pt|/(|Pt| + β|P⁄t| + α|t⁄P|)(3)

where α and β control the magnitude of penalties for FNs and FPs, respectively. Hence, the finally used Tversky loss function is defined as follows using Formula (4) [24]:

(4) $$L_{T}\left(\alpha ,\beta \right)=\frac{\sum_{i=1}^{N}p_{0i}v_{0i}}{\sum_{i=1}^{N}p_{0i}v_{0i}+\beta \sum_{i=1}^{N}p_{0i}v_{1i}+\alpha \sum_{i=1}^{N}p_{1i}v_{0i}}$$

In the above equation, *p*_0*i*_ and *p*_1*i*_ are the probability of voxel *i* lesion and non-lesion, respectively. Additionally, *v*_0*i*_ is 1 for a lesion and 0 for a non-lesion voxel and vice versa for the *v*_1*i*_.

Since image segmentation accuracy and robustness depend on choosing and use of DL models along with optimal training parameters, to more accurately and robustly segment disease infection areas or blobs depicting on chest CT images, we developed, tested, and compared five models based on AR-UNet with different training parameters, as summarized in Table 1. Additionally, based on the hypothesis that if the five models contain complementary prediction scores of pixels belonging to a disease infected area, the fusion of the predictions of all five selected models can further improve image segmentation results (i.e., prevent under-segmentation as much as possible). While involving several models comes with a longer processing time, the more reliable and precise prediction is worth the extra time. For each of these models, we have used Adam optimizer with a learning rate of 0.01.

#### 2.3.3. Segmentation of GGO and Consolidation Patches

Moreover, besides the overall infected region segmentation, it is of great importance to distinguish between different stages of COVID-19-infected pneumonia developments in the lung and provide better assistance to radiologists to assess disease severity levels. The “COVID-19 CT segmentation dataset” provides manual annotations with 3 infection types, the ground glass opacity (GGO), pleural effusion, and consolidation. Since the pleural effusion type is not of great interest in this study, we only included the GGO and consolidation labels in the training dataset.

**Figure 4:**
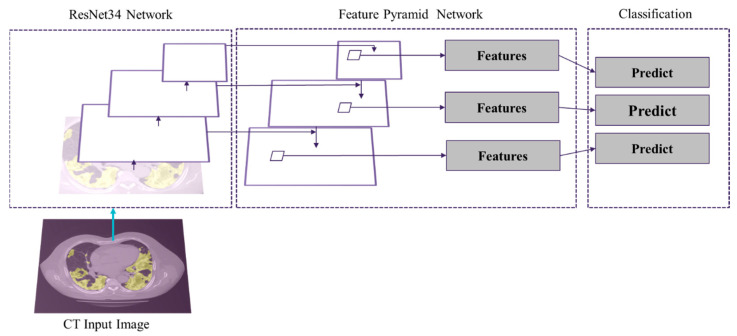
Overview of deep learning architecture for the patch segmentation model.

Like the infection region segmentation model, we tested various neural network architectures and hyperparameters aiming to achieve the best predictions. We applied a FPN model to categorize different stages of the COVID-19 in the infected area. This model has 23,915,590 trainable parameters. As depicted in Figure 4, the patch segmentation is based on Residual-Network (ResNet) and FPN model. ResNet34 is the backbone, and FPN is the feature extractor network. The loss function for this model is the categorical cross entropy which computes the cross entropy between the labels and predictions. This loss function is common when there are two or more label classes.

Although the staging model tends to over-segment the GGO regions, the consolidation segmentation is very accurate. To prevent the over-segmentation of the GGO area, the infection segmentation model is used to constrain the staging model. This model classifies each patch to three classes of normal tissue background, GGO, and consolidation.

#### 2.3.4. Integrated Model and GUI

In summary, three common deep neural network architectures were trained and employed in this study. For lung segmentation, we applied a publicly available model for lung parenchyma segmentation based on the UNet model. Additionally, an ensembled AR-UNet was developed for infection segmentation since the attention blocks have been shown to be very beneficial in image segmentation [22]. Moreover, an FPN model was applied to categorize the severity of the COVID-19 infected area. For each model, many parameters were tested and modified, including loss functions, fixed and variable learning rates, different encoders and decoders, and dropout rates. All models are written in Python, and the TensorFlow library is used to train and test the models.

After extracting the lung and infected lesions by the two segmentation models, the percentage of the infected lung volume is reported along with the average Hounsfield units (HU) inside the infected region, which can indicate the density of the lesion of interest and hence the severity of infection. This information is reported for the left and right lungs for each CT slice as well as the whole CT.

**Figure 5:**
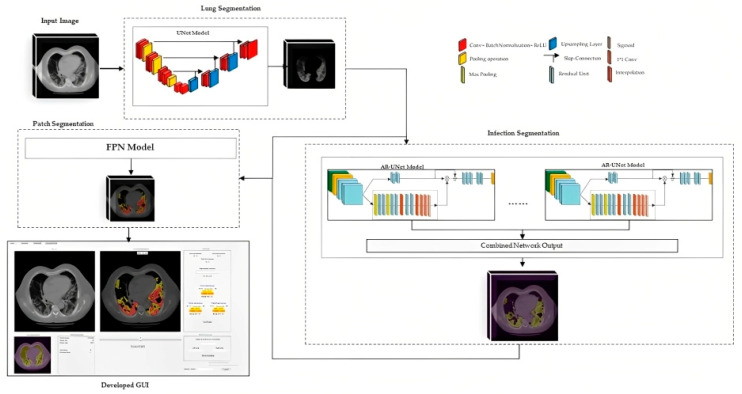
The flowchart of the proposed method.

Finally, to assist radiologists in the diagnosis of COVID-19 infected pneumonia using the DL model generated quantitative results or predictive scores, we also designed a stand-alone graphical user interface (GUI) as an interactive “visual-aid” tool, which can be installed on any Windows-based computers without the need for any specific programing language or library. Figure 5 illustrates the flow diagram of the developed DL model method and GUI tool.

### 2.4. Image Postprocessing and Correction

**Figure 6:**
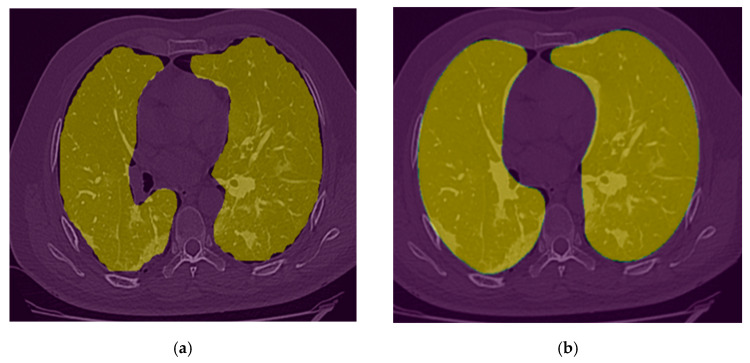
(**a**) Lung segmentation mask; (**b**) Post-processing lung segmentation.

After observing the output of the lung segmentation model, it was noted that in several cases with severe disease infection, a small percentage of the lung may be missing from the segmentation as shown in Figure 6a, which typically represents the disease infection area. To recover the missed lung area if the lung segmentation error is visually observed from our GUI, the user (i.e., radiologist) can call a specially-designed image post-processing function that applies a unique conventional image processing algorithm inspired by the rolling ball algorithm [27] to automatically correct segmentation error. This algorithm starts with extracting the lung contours followed by several steps and morphological filters such as disk drawing, filling holes, median, and erosion operations. As shown in Figure 6, it can convert a jagged and rough lung boundary, as shown in Figure 6a, to a smooth one that covers the previously missed lung area, as shown in Figure 6b. While it might lead to a small over-segmentation in some cases, the previously missed area contains very important infected lesions that can significantly affect the assessment of severe cases.

### 2.5. Evaluation

To evaluate new DL model performance, the model was first tested “as is” using an independent testing dataset of 80 CT scans. Next, we asked two expert chest radiologists to retrospectively read and review these 80 sets of CT images. Each radiologist read and examined half of the CT scans (40 patients) and reported the patient infection spread in percentage based on their judgment of the percentage of infected lung volume. These subjectively assessed values were then collected and compared to the values generated by the DL model. It is important to note that in this new testing image dataset of 80 clinical cases, there are no manually annotated lung and disease infection area segmentation marks. Thus, no Dice coefficients can be computed, and we only compared the agreement between the radiologists and the DL model in predicting the percentage of disease infected lung area (or volume) based on the predicted result of infection area ratio or spread scores between radiologists’ assessment and DL models.

Moreover, in order to test radiologists’ confidence level to accept DL-generated infection area segmentation results, we showed radiologists the DL segmentation results displayed on the developed GUI and asked them to rate their acceptance level of the infection area segmentation of each CT slice with a score of 1 (poor segmentation) to 5 (excellent segmentation).

Last, we asked the radiologists to assign each patient to the group of mild infection cases that are dominated by GGO or the group of severe infection cases that have a significant fraction of consolidation areas or blobs. We then compared the agreement between the DL model generated case classification results and the radiologists’ classification results. A corresponding confusion matrix was generated for the comparison and diagnostic accuracy computation.

## 3. Results

**Figure 7:**
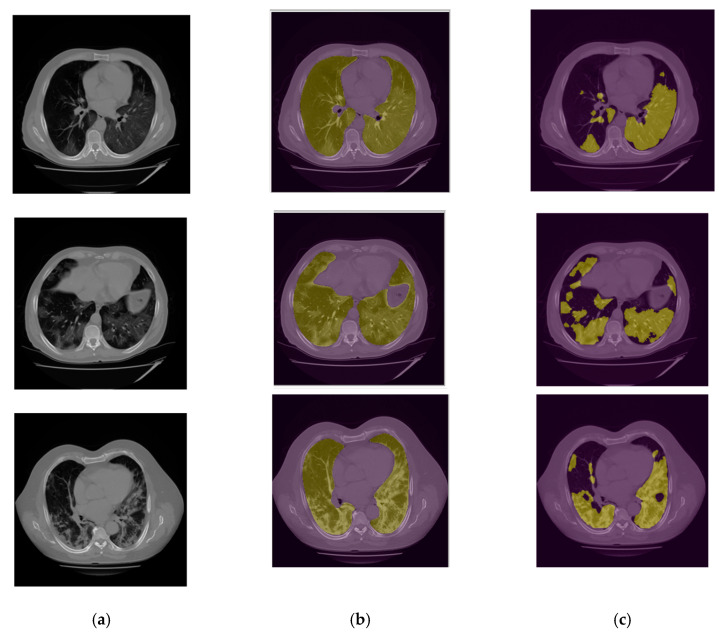
(**a**) Raw CT image; (**b**) Lung mask; (**c**) Infection Segmentation.

**Figure 8:**
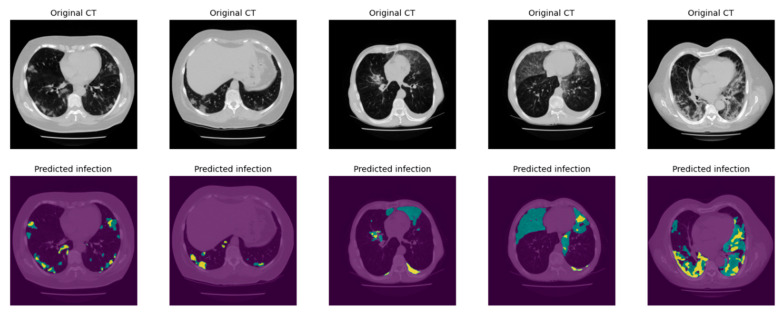
Patch segmentation results. The green area represents the GGO and Crazy Paved pattern. The yellow area shows the Consolidation area.

Figure 7 shows several image examples of DL-model generated lung and infection segmentation results. The left column illustrates the raw CT images, while the second and third columns illustrate the masks of the segmented lung and disease infection areas, respectively. In addition, Figure 8 shows the patch segmentation results of GGO and consolidation areas (or blobs), respectively. By using the commonly used evaluation index in image segmentation namely, the intersection over union (IOU), the quantitative data analysis results show that IOUs are 0.78 and 0.88 for the disease-infection region segmentation model and for the patch model, respectively.

**Figure 9:**
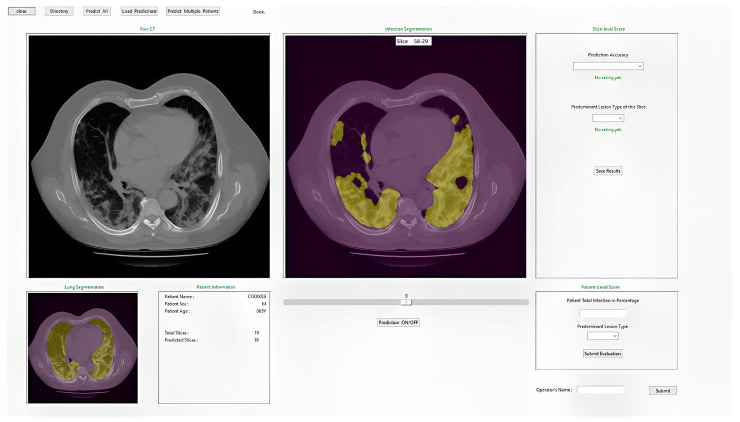
Illustration of the developed GUI for lung and COVID-19 infection segmentation.

Figure 9 shows a snapshot of the GUI window used in this study to obtain the subjective ratings from the radiologists. Using this GUI tool, radiologists can observe the raw CT image and the predicted segmentation side by side for better comparison. The radiologists can also rate the accuracy or acceptance level of the DL-generated disease infection area segmentation on each slice using a rating scale from 1 to 5, as well as provide their overall assessment of lung infection spread. Additionally, the lung segmentation is also visualized to make sure that the predicted spread scores are reliable. If a significant portion of the lung is missing, the radiologist can call and run the function to correct the segmentation errors as described in the Methods section of this paper.

**Figure 10:**
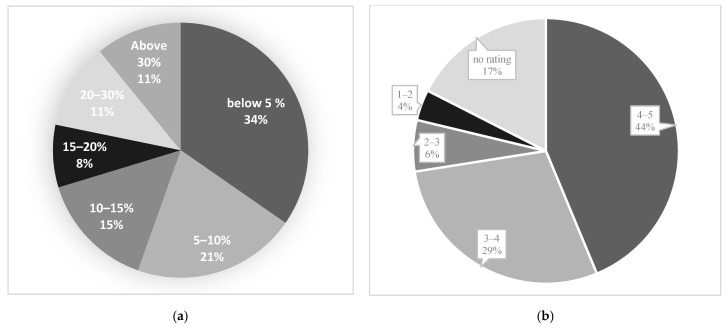
Part (**a**) illustrates the difference between the spread score of radiologists and the predicted score by the model; part (**b**) presents the average ratings of radiologists on the test dataset.

Figure 10 shows two diagrams that illustrate the distribution of our data analysis results to compare the agreement between the DL-model and radiologists in segmentation or estimation of disease-infected volumes, and acceptance level by radiologists of DL model generated disease region segmentation results. From these two summary or comparison diagrams, we observe the following study results.

(1) From Figure 10a, we observe that in 34% (27/80) of testing cases, the difference between the DL model generated diseased region segmentation and radiologist’s estimation is less than 5% (indicating the accuracy > 95%).
(2) In 55% (44/80) of testing cases, the difference between the DL model generated diseased region segmentation and radiologist’s estimation is less than 10% (or accuracy > 90%).
(3) In 90% (72/80) of testing cases, the difference between the DL model generated diseased region segmentation and radiologist’s estimation is less than 30% (or accuracy > 70%).
(4) From Figure 10b, we observe that in 73% (58/80) of testing cases, radiologists rated a score of 3 or higher indicating an acceptable lung and disease-infection region segmentation results generated by the DL model.

Additionally, the ratings of the testing cases with high spread score accuracy have been carefully analyzed to ensure that the high accuracy is not by chance. For example, among the testing cases with more than 95% spread accuracy, the radiologists rated an acceptance score higher than 3 in over 78% of cases, and among the testing cases with >90% accuracy, 84% of cases received an acceptance rating higher than 3 indicating the DL segmentation is acceptable, and the spread score is reliable.

Moreover, to evaluate the performance of our DL model in identifying different stages of COVID-19, the radiologists also put a label on the infected regions. Then, the results of our model and radiologists were compared together. Table 2 shows the confusion matrix of the disease staging performance. When using radiologists’ rating or disease level classification results as a reference (“ground-truth”), our DL model yields an 85% (68/80) accuracy in predicting or classifying disease infection severity levels in this testing dataset.

## 4. Discussion

In the last three years, large number of studies have been reported in the literature to develop DL-based models of detection and classification of COVID-19 infected pneumonia using chest X-ray radiographs and/or CT images. However, as reported in a comprehensive review study [16], no previous DL model was accepted in clinical practice to effectively assist radiologists. To effectively address or solve this challenge and make the DL model acceptable to radiologists, we conducted a unique model development and observer-involved comparison study. This study has the following unique characteristics and/or new observations.

First, we tested a new hypothesis to quantify percentages of COVID-19 infected volume and demonstrated a potential application of a novel DL model in the segmentation of the COVID-19 generated pneumonia infection in chest CT images. One of the innovations of this study is that we developed a combined five AR-UNet models for the infected region segmentation and a novel lung segmentation correcting algorithm based on conventional image processing techniques to ensure all infected lesions are included in the prediction. Furthermore, we applied an FPN model to identify different stages of the COVID-19 infected area.

Second, since physicians including radiologists have low confidence in accepting results generated by current “black box” type artificial intelligence (AI) or DL models, developing “explainable AI” tools [28] has been attracting broad research interest in the medical imaging field. Thus, we designed and implemented a graphic user interface (GUI) as an interactive “visual-aid” tool (Figure 9) that shows DL segmented disease infection areas. This stand-alone GUI allows radiologists to easily navigate through all generated outputs, rate each CT slice automatic segmentation, and submit their assessment of the percentage of lung volume with COVID-19 infection. Additionally, the radiologist can also call a supplementary image postprocessing algorithm to automatically correct the possibly identified segmentation errors. Our experience and results of the observer reading and preference study demonstrate that using this interactive GUI-based “visual-aid” supporting tool can provide radiologists with the reasoning of DL model generated prediction results and thus increase their confidence to use the DL model in their decision-making process of disease diagnosis.

Third, based on our interaction with the radiologists, we learned that radiologists typically assign the patients into 3 classes of disease severity, namely, mild, moderate, and severe diseases, based on the distribution or domination of GGO, pleural effusion, and consolidation patterns. Thus, we believe that to increase its clinical utility, the DL model should also have a function or capability to assign each testing case to one of these three classes. Since in three image datasets used in this study, very few pleural effusion patterns exist, we developed a patch segmentation-based model to identify GGO and consolidation areas depicted on each CT image slice and then predict or classify the cases into either mild/moderate (A) and severe (C) classes as shown in Table 2. In this way, we were able to compare disease severity prediction results between the radiologists and DL model. In future studies, we need to collect more study cases with more diversity. Thus, we can apply the same DL concept to train the model that enables us to classify 3 classes of disease severity.

Fourth, we conducted a unique observer reading and preference study involving two chest radiologists and reported data comparison results. Thus, unlike many previous studies in this field, which only reported Dice coefficients of agreement between DL model generated image segmentation results and the manual segmentation results of one radiologist, which does not have a real clinical impact due to the large inter-reader variability in manual image segmentation or annotation, we used a simple and more efficient or practical method to evaluate DL model segmentation results by asking radiologists to rate the acceptance level of DL model segmentation using a 5 rating scale. This practical approach has proved quite effective and higher clinically relevant in the medical imaging field [29]. Our study generates quite encouraging results or observations of the higher agreement between the DL-model generated segmentation and radiologists’ estimation of the COVID-19 infected region or volume, as well as the higher acceptance rate of radiologists to the DL model-segmented results (Figure 10).

The above observations also demonstrate a new contribution of this study, which provides the research community with new scientific data or evidence. (1) Our study demonstrates a higher acceptance rate of radiologists to DL model generated results of disease-infected region segmentation. This supports the feasibility of improving the efficacy of radiologists in reading CT images to diagnose disease because the DL model can not only replace the tedious and time-consuming process of subjectively estimating the percentages of the pneumonia regions or volume, but also avoid or reduce the large inter-reader variability. (2) Our study also supports the importance of future evaluation studies to better investigate and find the optimal interaction between DL models and radiologists to reduce the application gaps and facilitate the process to make DL models or technology clinically useful or acceptable tools in future clinical practice. (3) Although this study only used COVID-19 cases to segment and quantify pneumonia regions or volume, if successful, the demonstrated new DL model and evaluation approach can be easily adapted to segment and quantify other types of virus infection pneumonia or other interstitial lung diseases (ILD) in future research studies.

Last, we also recognize the limitations of this study, including the small image datasets and involving only two radiologists. Thus, this is a very preliminary study. The developed DL model along with the GUI tool needs to be further optimized and validated using large and diverse image cases. We also need to recruit more radiologists to evaluate model performance and potential clinical utility in future studies. Despite the limitations, we believe that this is a unique and valid study.

## 5. Conclusions

In this study, we developed a new ensembled DL model to automatically segment and quantify the COVID-19 infected pneumonia region or volume and predict disease severity level. To increase the model transparency and radiologists’ confidence in considering or accepting DL model generated results, we designed and integrated an interactive GUI as a “visual aid” tool to the DL model. The most important novelty or contribution of this study is that we conducted a unique observer reading and preference study. The data analysis and comparison results demonstrate the higher agreement between DL model and radiologists in disease region segmentation or estimation and disease severity level prediction. However, this is a preliminary and concept-approval type study. More evaluation studies involving more radiologists and more diverse image cases are needed in future research. If successful, such DL-based disease quantification models with interactive visual-aid tools will have promising potential to provide radiologists with useful decision-making supporting tools to improve the accuracy of lung disease diagnosis in future clinical practice.

## Figures and Tables

**Table 1:** The detail of the ensembled model for infection detection.

	Loss Function	Augmentation	Dropout
Model 1	Binary Cross Entropy	5 times	0
Model 2	Tversky	10 times	0
Model 3	Tversky	10 times	0.10
Model 4	Binary Cross Entropy	10 times	0
Model 5	Binary Focal Loss	5 times	0.10

**Table 2:** Confusion matrix illustrating the developed model’s stage detection. The cases dominated with GGO and crazy paved pattern area are classified as “A” group, and “C” represents the cases with significant consolidation area (blobs).

Radiologists\Model	A	C
A	61	2
C	10	7

## Data Availability

Publicly available datasets were analyzed in this study. This data can be found here: [https://www.kaggle.com/andrewmvd/covid19-ct-scans] (accessed on 17 May 2021), [https://coronacases.org/] (accessed on 17 May 2021), [https://radiopaedia.org/] (accessed on 17 May 2021).

## References

[1] Müller N.L., Staples C.A., Miller R.R., Abboud R.T. (1988). “Density mask”: An objective method to quantitate emphysema using computed tomography. Chest.

[2] Karimi R., Tornling G., Forsslund H., Mikko M., Wheelock M., Nyrén S., Sköld C.M. (2014). Lung density on high resolution computer tomography (HRCT) reflects degree of inflammation in smokers. Respir. Res..

[3] Ciotti M., Ciccozzi M., Terrinoni A., Jiang W.C., Wang C.B., Bernardini S. (2020). The COVID-19 pandemic. Crit. Rev. Clin. Lab. Sci..

[4] Heidari M., Mirniaharikandehei S., Khuzani A.Z., Danala G., Qiu Y., Zheng B. (2020). Improving the performance of CNN to predict the likelihood of COVID-19 using chest X-ray images with preprocessing algorithms. Int. J. Med. Inform..

[5] Fan D.-P., Zhou T., Ji G.-P., Zhou Y., Chen G., Fu H., Shen J., Shao L. (2020). Inf-net: Automatic COVID-19 lung infection segmentation from CT images. IEEE Trans. Med. Imaging.

[6] Wang S., Kang B., Ma J., Zeng X., Xiao M., Guo J., Cai M., Yang J., Li Y., Meng X. (2021). A deep learning algorithm using CT images to screen for Corona Virus Disease (COVID-19). Eur. Radiol..

[7] Porwal P., Pachade S., Kokare M., Deshmukh G., Son J., Bae W., Liu L., Wang J., Liu X., Gao L. (2020). IDRid: Diabetic retinopathy—Segmentation and grading challenge. Med. Image Anal..

[8] Shi T., Jiang H., Zheng B. (2022). C2MA-Net: Cross-modal cross-attention network for acute ischemic stroke lesion segmentation based on CT perfusion scans. IEEE Trans. Biomed. Eng..

[9] Jones M.A., Islam W., Faiz R., Chen X., Zheng B. (2022). Applying artificial intelligence technology to assist with breast cancer diagnosis and prognosis prediction. Front. Oncol..

[10] Islam W., Jones M., Faiz R., Sadeghipour N., Qiu Y., Zheng B. (2022). Improving performance of breast lesion classification using a ResNet50 model optimized with a novel attention mechanism. Tomography.

[11] Wu Y.-H., Gao S.-H., Mei J., Xu J., Fan D.-P., Zhang R.-G., Cheng M.-M. (2021). JCS: An explainable COVID-19 diagnosis system by joint classification and segmentation. IEEE Trans. Image Process..

[12] Abbas A., Abdelsamea M.M., Gaber M.M. (2021). 4S-DT: Self-supervised super sample decomposition for transfer learning with application to COVID-19 detection. IEEE Trans. Neural Netw. Learn. Syst..

[13] Ozturk T., Talo M., Yildirim E.A., Baloglu U.B., Yildirim O., Acharya U.R. (2020). Automated detection of COVID-19 cases using deep neural networks with X-ray images. Comput. Biol. Med..

[14] Zhuang Y., Rahman M.F., Wen Y., Pokojovy M., McCaffrey P., Vo A., Walser E., Moen S., Xu H., Tseng T.L. (2022). An interpretable multi-task system for clinically applicable COVID-19 diagnosis using CXR. J. X-Ray Sci. Technol..

[15] Clement J.C., Ponnusamy V., Sriharipriya K.C., Nandakumar R. (2022). A survey on mathematical, machine learning and deep learning models for COVID-19 transmission and diagnosis. IEEE Rev. Biomed. Eng..

[16] Roberts M., Driggs D., Thorpe M., Gilbey J., Yeung M., Ursprung S., Aviles-Rivero A.I., Etmann C., McCague C., Beer L. (2021). Common pitfalls and recommendations for using machine learning to detect and prognosticate for COVID-19 using chest radiographs and CT scans. Nat. Mach. Intell..

[17] Tian X., Huang R.Y. (2020). Standardization of imaging methods for machine learning in neuro-oncology. Neuro-Oncol. Adv..

[18] Simard P.Y., Steinkraus D., Platt J.C. Best practices for convolutional neural networks applied to visual document analysis. Proceedings of the Seventh International Conference on Document Analysis and Recognition, IEEE.

[19] Buslaev A., Iglovikov V.I., Khvedchenya E., Parinov A., Druzhinin M., Kalinin A.A. (2020). Albumentations: Fast and flexible image augmentations. Information.

[20] Ronneberger O., Fischer P., Brox T. (2015). U-net: Convolutional networks for biomedical image segmentation. International Conference on Medical Image Computing and Computer-Assisted Intervention.

[21] Lin T.Y., Dollár P., Girshick R., He K., Hariharan B., Belongie S. Feature pyramid networks for object detection. Proceedings of the IEEE Conference on Computer Vision and Pattern Recognition.

[22] Oktay O., Schlemper J., Folgoc L.L., Lee M., Heinrich M., Misawa K., Mori K., McDonagh S., Hammerla N.Y., Kainz B. (2018). Attention u-net: Learning where to look for the pancreas. arXiv.

[23] Yakubovskiy P. (2019). Segmentation Models.

[24] Salehi S.S.M., Erdogmus D., Gholipour A. (2017). Tversky loss function for image segmentation using 3D fully convolutional deep networks. International Workshop on Machine Learning in Medical Imaging.

[25] Li C., Liu Y., Yin H., Li Y., Guo Q., Zhang L., Du P. Attention residual U-Net for building segmentation in aerial images. Proceedings of the 2021 IEEE International Geoscience and Remote Sensing Symposium IGARSS.

[26] Wang Q., Ma Y., Zhao K., Tian Y. (2022). A comprehensive survey of loss functions in machine learning. Ann. Data Sci..

[27] Park S.C., Tan J., Wang X., Lederman D., Leader J.K., Kim S.H., Zheng B. (2011). Computer-aided detection of early interstitial lung diseases using low-dose CT images. Phys. Med. Biol..

[28] Arrieta A.B., Díaz-Rodríguez N., Del Ser J., Bennetot A., Tabik S., Barbado A., Garcia S., Gil-Lopez S., Molina D., Benjamins R. (2020). Explainable Artificial Intelligence (XAI): Concepts, taxonomies, opportunities and challenges toward responsible AI. Inf. Fusion.

[29] Pu J., Leader J.K., Zheng B., Knollmann F., Fuhrman C., Sciurba F.C., Gur D. (2008). A computational geometry approach to automated pulmonary fissure segmentation in CT examinations. IEEE Trans. Med. Imaging.

